# Intramyocardial transplantation of cardiac telocytes decreases myocardial infarction and improves post-infarcted cardiac function in rats

**DOI:** 10.1111/jcmm.12259

**Published:** 2014-03-21

**Authors:** Baoyin Zhao, Zhaofu Liao, Shang Chen, Ziqiang Yuan, Chen Yilin, Kenneth KH Lee, Xufeng Qi, Xiaotao Shen, Xin Zheng, Thomas Quinn, Dongqing Cai

**Affiliations:** a Key Laboratory for Regenerative Medicine, Ministry of Education, Ji Nan UniversityGuangzhou, China; b Joint Laboratory for Regenerative Medicine, Chinese University of Hong Kong-Ji Nan UniversityGuangzhou, China; c International Base of Collaboration for Science and Technology (JNU), The Ministry of Science and Technology & Guangdong ProvinceGuangzhou, China; d Department of Developmental and Regenerative Biology, Ji Nan UniversityGuangzhou, China; e Department of Molecular Genetics, Albert Einstein College of MedicineNew York, NY, USA; f Stem cell and Regeneration TRP, School of Biomedical Sciences, Chinese University of Hong KongHong Kong

**Keywords:** cardiac telocytes, myocardial infarction, post-infarction cardiac function

## Abstract

The midterm effects of cardiac telocytes (CTs) transplantation on myocardial infarction (MI) and the cellular mechanisms involved in the beneficial effects of CTs transplantation are not understood. In the present study, we have revealed that transplantation of CTs was able to significantly decrease the infarct size and improved cardiac function 14 weeks after MI. It has established that CT transplantation exerted a protective effect on the myocardium and this was maintained for at least 14 weeks. The cellular mechanism behind this beneficial effect on MI was partially attributed to increased cardiac angiogenesis, improved reconstruction of the CT network and decreased myocardial fibrosis. These combined effects decreased the infarct size, improved the reconstruction of the LV and enhanced myocardial function in MI. Our findings suggest that CTs could be considered as a potential cell source for therapeutic use to improve cardiac repair and function following MI, used either alone or in tandem with stem cells.

## Introduction

The reconstruction of damaged myocardium is still a major challenge on regenerative medicine [1–4]. The use of cell therapy for MI has been reported to improve cardiac function and reduce the infarct size [5–12]. However, recent research that includes more stringent placebo control and randomized trials has questioned whether cell therapy really functionally improves cardiac, especially the mid- and long-term therapeutic effects after MI in both animals and humans [13–21]. To date, the promise of complete cardiac regeneration has still not yet been achieved. Recent studies have shown that the supporting niche cells within cardiac units of the myocardium might play an important role in myocardial regeneration [22]. A novel cardiac interstitial cell, named telocyte, has recently been identified in the heart interstitium [23–30]. Within the cardiac stem cell niche, CTs might play an essential supporting role in nursing cardiac stem cells and the angiogenic cells in the myocardium. In this context, the cardiac telocytes might also play an important role in myocardial regeneration [31]. Recently, we reported that CT is one of the most consistent cell types in the cardiac unit and it is structurally and functionally important for the myocardium. Transplantation of CTs in acute myocardial infarction was able to decrease the infarct size and improve the myocardial function [32]. However, the midterm effect of CT transplantation after MI and the cellular mechanisms behind CT*s beneficial effects are not understood. The present study was designed to address these intriguing issues.

## Materials and methods

### Animal

Three-month-old female Sprague-Dawley (SD) rats (250–300 g) were utilized in the present study. The rats were housed for 2 weeks to allow them to adapt before experimentation. They were provided with food and water *ad libitum*. Animal care, surgery and handling procedures were performed according to regulations set by The Ministry of Science and Technology of the People*s Republic of China [(2006) 398] and approved by Ji Nan University Animal Care Committee.

### Myocardial infarction studies

Three-month-old female SD rats (*n* = 4–5 for each group) were used to generate myocardial infarction as previously described [33]. Briefly, the rats were anaesthetized with ketamine (100 mg/kg, ip) and underwent a left intercostal thoracotomy. The left anterior descending coronary artery (LAD) was identified and then the vessel was ligated directly below the left atrial appendage, with gauged 8-0 nylon sutures. The presence of pallor and abnormal movement of the LV confirmed LAD occlusion. The chest wall was then closed, the lungs were inflated, the rat was extubated and the tracheotomy was closed. After recovery, the rats were returned to the animal facility for 14 weeks. At the end of the study, the rats were scarified and the hearts were harvested. They were fixed in 4% paraformaldehyde, embedded in paraffin wax and section. Some of the sections were prepared for Masson*s trichrome staining.

### Isolation of cardiac telocytes

Young (3-month-old) SD female rats were used for the isolation of CT as previously described [32]. Briefly, the hearts of the rats (female, 3-month-old) were minced, and then the tissues were treated with 2.5 ml of DMEM+ 0.05% collagenase P (Roche, Branchburg, NJ, USA) and 0.1% trypsin (Amresco, Solon, OH, USA) at 37°C on a shaker (180 r.p.m.) for 15 min. After the suspension was removed, trypsin medium was added and the mixture was incubated at 37°C on a shaker for 45 min. The digested tissue was dissociated by pipetting gently every 15 min. The supernatant was then filtered through a 100-μm and then a 41-μm nylon mesh (Millipore, Billerica, MA, USA), and the collected cell suspension was centrifuged at 50 × g for 2 min. The supernatant was then removed and re-centrifuged at 300 × g for 10 min. The pellet was re-suspended in 5 ml of PEB medium [PBS supplemented with 0.5% bovine serum albumin. and 2 mM EDTA (pH = 7.2)]. The mixture was then centrifuged at 38 × g for 2 min to remove the debris, and the collected supernatant was further centrifuged at 200 × g for 10 min. The cell pellet was then mixed with 1 ml of PEB and 5 ml of rabbit anti-rat c-kit antibody (1:200; cat no. NBP1-19865; Novus, Littleton, CO, USA), and the sample was incubated at 4°C for 40 min. An additional 2 ml of PEB was then added, and the mixture was centrifuged at 458 × g for 4 min to collect the cells. The pellet was re-suspended in 160 ml of PEB, and 20 ml of a solution containing magnetic beads (goat anti-rabbit lgG-microbeads, cat. no. 5111007039; Miltenyi Biotec) was added, followed by incubation at 4°C for 25 min. The mixture was next added to an MS column (Miltenyi Biotec, Bergisch Gladbach, Germany) in a magnetic field, and the unlabelled cells were allowed to pass through. The MS column was then removed from the magnetic field, and the labelled cells were flushed out with PEB. The isolated cell pellet was collected after centrifugation at 458 × g for 4 min. It was established that by using the method, more than 93% of the isolated cells were c-kit^+^ and CD34^+^. Only passage 3 or less isolated CTs were used for experimentation.

### Transplantation of cardiac telocytes

The potential therapeutic effects of CT in MI were investigated. Three different sets of 3-month-old female SD rats were produced following MI. The rats were either injected with CTs (*n* = 5), c-kit^−^ cells (*n* = 4) or PBS (*n* = 5, control) as previously described [33]. The intramyocardial injections were performed within 30 min after the LAD ligation. Approximately 10^6^ of CTs in PBS, 10^6^ of c-kit^−^ cells in PBS or PBS alone were injected, using a 30-gauge needle inserted on a 100 μl Hamilton syringe. A total of five injections (10 μl/injection) per heart were made, with three injections in border area of ischaemic zone and two injections in the centre of the ischaemic area. The chest wall was then closed, the lungs inflated, extubated and the tracheotomy was closed. After recovery, the rats were returned to the animal holding facility for 14 weeks.

### Analysis of the infarct size, wall thickness of border zone and thickness of infarct myocardium

The extent of myocardial infarction measured, at the level of the mid-papillary heart muscles, was scored following Masson*s trichrome staining. The images were analysed using an Image J1.22 software (NIH Image). The Infarction size, with linear approximations to account for area gaps in histology, was expressed as a percentage of the total LV myocardial area as previously described [33]. The wall thickness of the border zone of LV (WTBZ) and the thickness of infarcted myocardium of the LV (TIM) are schematically illustrated in Figure 7A. Both structures were measured with our Image J 1.22 software. The measurements and quantifications were conducted in a double-blind fashion by two independent investigators.

### Echocardiography

Transthoracic echocardiograms were performed and recorded in rats, 14 weeks after LAD ligation and transplantation of CT. The experimental rats were anaesthetized with ketamine (100 mg/kg, ip). The echocardiographic parameters were then collected using an Acuson Sequoia 256c ultrasound system equipped with a 13-MHz linear transducer of Vevo 770 echocardiogram (VisualSonics, Toronto, Canada). Briefly, the anterior chest wall was shaved, and the rat was placed in a left lateral decubitus position. A rectal temperature probe was inserted and the body temperature was carefully maintained between 37 and 37.5°C on a heating pad throughout the study. The parasternal long-axis, parasternal short-axis and two apical four-chamber views were used to obtain 2D-M-mode. The systolic and diastolic anatomic parameters were obtained from M-mode tracings at the mid-papillary level. The ejection fraction (EF) was calculated by the area-length method [34,35].

### Semi-quantification of c-kit/CD34-positive cells

Representative sections, obtained from the level of the mid-papillary heart muscles, were used for double immunofluorescent staining. Briefly, the sections were first treated with 0.1% pronase (cat no. 10512; Yuanye, Shanghai, China) in Tris-HCl buffer (pH = 7.4) at 37°C for 15 min. for antigen retrieval. They were then washed three times with PBS (pH = 7.4) and incubated with goat anti-rat-CD34 antibody (1:500; cat no. AF4117; R&D, Minneapolis, MN, USA) at 4°C overnight. FITC-donkey anti-goat IgG was then added to the section and further incubated for 1 hr. Subsequently, rabbit anti-rat c-kit antibody (1:300; cat no. NBP1-19865; Novus) was added to the sections, and the sections were incubated at 4°C overnight. Then, CY3-goat anti-rabbit IgG was added, and the sections were incubated for 1 hr. The sections were then counter-stained with DAPI and mounted with mounting medium. In this study, we used c-kit^+^/CD34^+^ with a DAPI^+^ nucleus and a very small cell body (piriform/spindle/triangular) with an extremely long and thin prolongation (length ≥60 μm) to identify cardiac telocytes. Images (100×) of the infarcted zone were obtained using fluorescence microscopy (Leica, Wetzlar GmbH- DM4000B, Germany). The anti-c-kit/CD34 and DAPI images from the same field were merged using Leica cw4000 FISH software. The cardiac telocytes were counted using a double-blinded method. The cell density in infarct zone ± standard deviation was used in the semi-quantitative analysis. Three animals for each group were applied to analyse.

### Semi-quantification of angiogenesis

Immunohistological staining was performed on the heart samples using a rabbit vWF antibody (cat. no. F3520; Sigma-Aldrich, ST. Louis, MO, USA), peroxidase-conjugated goat anti-Rabbit IgG (cat. no. SA00001-2, Proteintech, Chicago, IL, USA) and a DAB-Plus staining Kit (cat. no. 00-2020; Life Technologies, Grand Island, NY, USA). vWF is a marker for endothelial cells and was used to evaluate the number of blood vessels present in the infarct zone and the border zone. Briefly, hydrated heart sections (obtained from the mid-papillary level) were first treated for 5 min in 0.5% hydrogen peroxide diluted in PBS, then washed in PBS and incubated for 1 hr in 1% BSA-blocking solution. The heart sections were incubated with vWF antibody (1:300) overnight at 4°C. After washing with three changes of PBS, the biotinylated secondary antibody was added for 1 hr. DAB was used as the chromagen. After staining, the sections were dehydrated, cleared in xylene and then mounted with mounting medium. The number of vWF^+^ blood vessels which present in the whole infarct zone and border zone was photographed and counted under microscope (20× magnification). The number of vessels present per mm^2^ was compared between the different groups. The measurements were conducted in a double-blinded fashion by two independent investigators.

### Semi-quantification of fibrosis in the MI heart

Sections of the experimental and control infracted hearts were stained using a modified Masson*s trichrome staining protocol (which excluded the Beibrich scarlet-acid fuchsin staining step) was used to reveal the presence of collagen. The fibrillar collagen was stained blue in the sections. Representative sections of the intact heart acquired from the mid-papillary level were stained and photographed. The area of blue staining in the infarct zone and non-infarct zone was indentified and measured using Image-Pro Analyzer 6.0 software. The perivascular collagen volume area (PVCA) was expressed as the ratio of peripheral collagen area surrounding the small artery-to-artery lumen area. The small arteries were selected for detail analysis at 40× magnification and the average was used as the definitive PVCA. The analysis and quantification were performed in a blinded fashion manner.

### Statistical analysis

All measurements are presented as mean ± standard deviation. The one-way anova test, LSD test and SPSS version-11 software were used to determine whether the control and experimental data were statistically significant (*P* < 0.05).

## Results

### Transplantation of CTs decreased the infarct size after MI

**Fig. 1:**
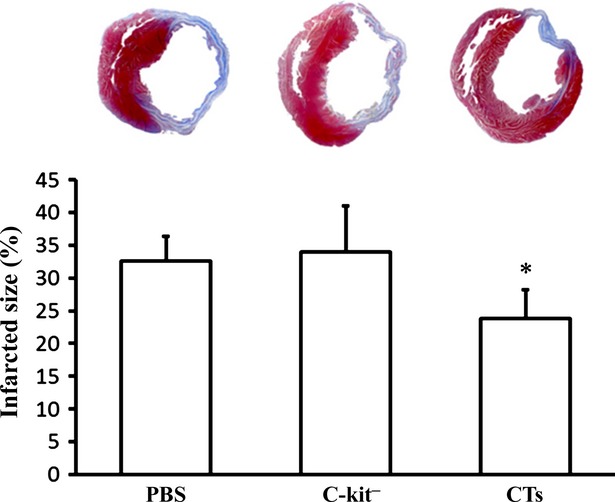
Transplantation of cardiac telocytes decreased the infarct size in myocardial infarction (MI). CTs (10^6^) were simultaneously injected into the infarcted area and the border zone after LAD ligation MI. The infarction size was identified by Masson*s trichrome staining and analysed 14 weeks after LAD ligation. The infarction size (%LV) in the CT-treated group was significantly smaller (*P* < 0.05) than the c-kit^−^ cell-treated group and PBS-treated group. *N* = 4–5 for each group. *P < 0.05, CT-treated group *versus* c-kit^−^ cell-treated group and PBS-treated group.

In the present study, the midterm therapeutic effect of CT implantation following MI was investigated. The cardiac telocytes (10^6^ cells) were simultaneously injected into the infarct site and the border zone after MI. The infarction size was analysed 14 weeks after LAD ligation. It was found that the infarction size (%LV) in the CT-treated group was significantly smaller than in c-kit^−^ cell-treated group and PBS-treated group (*P* < 0.05). However, the infarction size in the group that received the c-kit^−^ cells was similar to the PBS-treated group (*P* > 0.05; Fig. 1).

### Transplantation of CTs improved myocardial function after MI

**Fig. 2:**
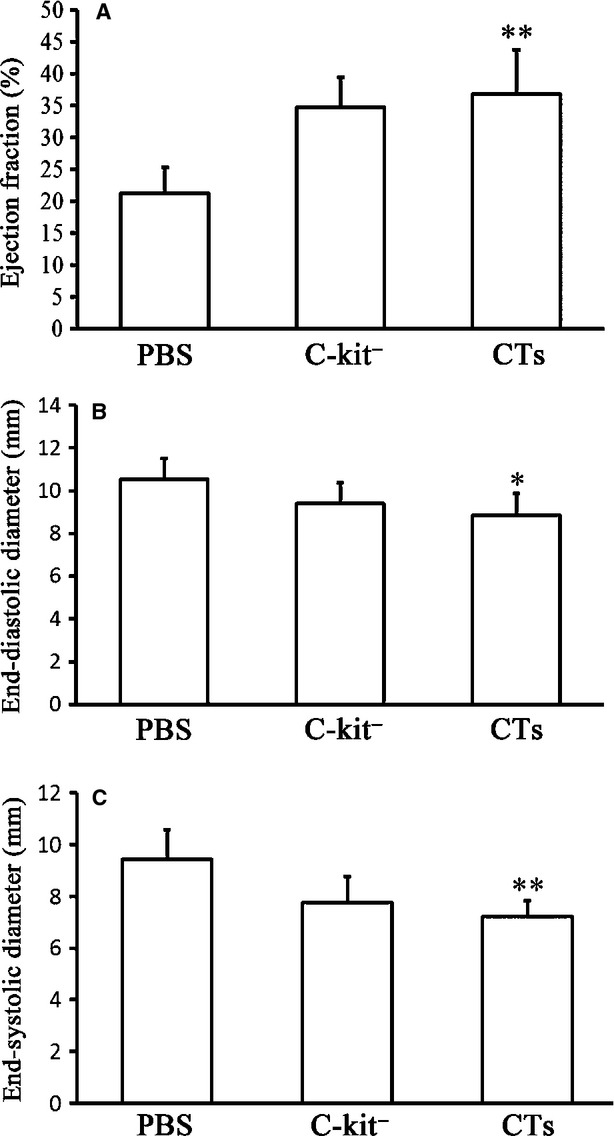
Transplantation of cardiac telocytes improved myocardial function in myocardial infarction (MI). Myocardial function of MI hearts was analysed by echocardiography 14-week post-LAD ligation showing (**A**) the ejection fraction of the CT-treated group was significantly higher than the PBS-treated group (*P* < 0.01). (**B**) the final diastolic diameters of the CT-treated group was significantly lower than the PBS-treated group (*P* < 0.05). (**C**) the final systolic diameters of the CT-treated group was significantly lower than the PBS-treated group (*P* < 0.01). *N* = 4–5 for each group. *P < 0.05, CT-treated group *versus*PBS-treated group; **P < 0.01, CT-treated group *versus*PBS-treated group.

The myocardial function was evaluated by echocardiography, 14 weeks after LAD ligation and CT transplantation. The results of echocardiography for the MI hearts revealed that the EF of the CT-treated group was significantly higher than the PBS-treated group (*P* < 0.01). The EF of the CT-treated group was higher than that of the c-kit^−^ cell-treated group, however, the difference was not statistically significant (*P* > 0.05; Fig. 2A). The final diastolic diameters of the CT-treated group were lower than those of the c-kit^−^ cell-treated group and PBS-treated group (*P* < 0.05 *versus* PBS-treated group; Fig. 2B). In addition, the final systolic diameters of the CT-treated group were lower than those of the c-kit^−^ cell-treated group and PBS-treated group (*P* < 0.01 *versus* PBS-treated group; Fig. 2C).

### Transplantation of CTs increased c-kit^+^/CD34^+^ cells in 14-week-old infarct site

**Fig. 3:**
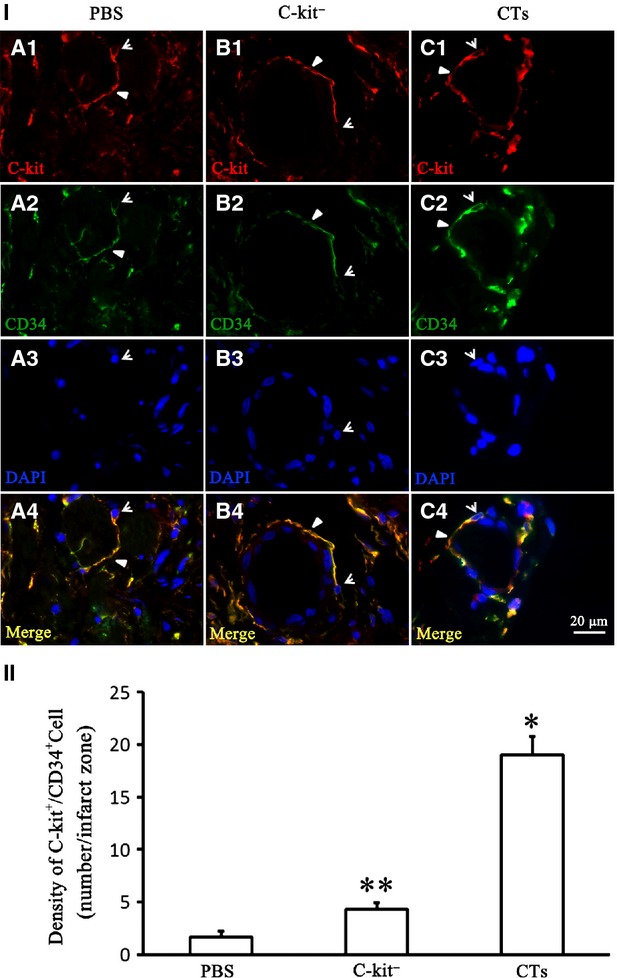
Transplantation of CTs increased c-kit^+^/CD34^+^ in 14-week-old infarct site. Double immunofluorescent staining for c-kit^+^/CD34^+^ cells was examined 14 weeks after transplantation. (I) Representative morphology of c-kit^+^/CD34^+^ CTs. (A_1-4_) PBS-treated group. (B_1-4_) c-kit^−^ cell-treated group; (C_1-4_) CT-treated group. C-kit^+^/CD34^+^ CTs were found near and surrounding the blood vessels in CT-treated group, c-kit^−^ cell-treated group and PBS-treated group. Open arrow: Cell body and/or nucleus of CT. Arrow: Telopode of CT; bar = 20 μm. (II) Semiquantitative image analysis of c-kit^+^/CD34^+^ CTs revealed that the density of c-kit^+^/CD34^+^ CTs in the CT-treated group was significantly higher than in the c-kit^−^ cell-treated group and PBS-treated group (*P* < 0.001). In addition, the density of c-kit^+^/CD34^+^ CTs in the c-kit^−^ cell-treated group was significantly higher than the PBS-treated group (*P* < 0.05), while the density of c-kit^+^/CD34^+^ CTs in CT-treated group was approximately fourfold higher than the c-kit^−^ cell-treated group (*P* < 0.001). *P < 0.001; **P < 0.05; *N* = 3.

To evaluate the possible effects for reconstruction CT network in the infarct zone, double immunofluorescent staining for the presence of c-kit^+^/CD34^+^ cells was performed 14 weeks after CT transplantation. It was found that some of c-kit^+^/CD34^+^ CTs were found near or surrounding the blood vessels in the CT-treated group, c-kit^−^ cell-treated group and PBS-treated group (Fig. 3I). Semi-quantitative image analysis revealed that the density of c-kit^+^/CD34^+^ CTs in the CT-treated group was significantly higher than that of the c-kit^−^ cell-treated group and PBS-treated group (*P* < 0.001). In addition, the density of c-kit^+^/CD34^+^ CTs in the c-kit^−^ cell-treated group was significantly higher than in the PBS-treated group (*P* < 0.05). The density of c-kit^+^/CD34^+^ CTs in the CT-treated group was approximately fourfold higher than in the c-kit^−^ cell-treated group (*P* < 0.001; Fig. 3II).

### Transplantation of CTs improved angiogenesis in MI

**Fig. 4:**
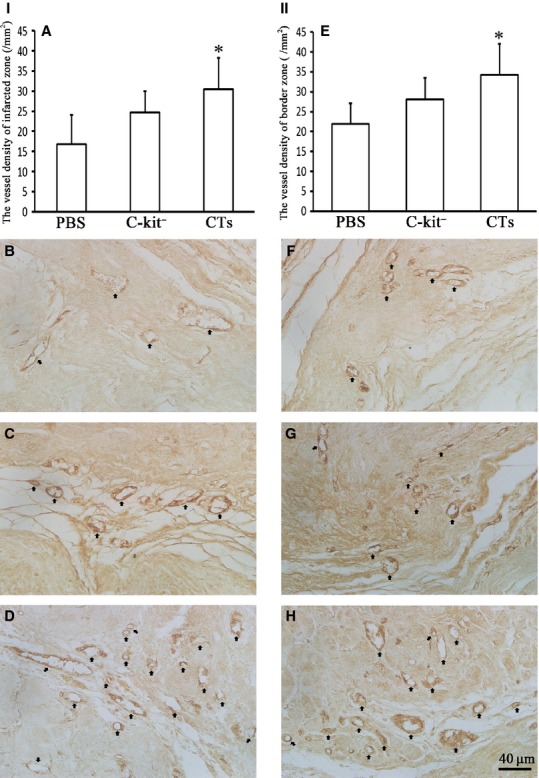
Transplantation of cardiac telocytes enhanced angiogenesis in myocardial infarction (MI). The density of vessels in MI hearts 14-week post-LAD ligation was determined by comparing the presence of vWF^+^ vessels in the infarct zone and border zone. (I) In the infarcted area, the vessel density of the CT-treated group was significantly larger than the PBS-treated group (*P* < 0.05). The vessel density of the c-kit^−^ cell-treated group appeared larger than the PBS-treated group, but was not statistically significant (*P* > 0.05). (**A**) Showing the semi-quantification analysis of vessel density. The representative morphologies of the PBS-treated group (**B**), the c-kit^−^ cell-treated group (**C**) and the CT-treated group (**D**). (II): In the border zone, the vessel density of CT-treated group was significantly larger than the PBS-treated group (*P* < 0.05). (**E**) showing the semi-quantification analysis of vessel density. The representative morphologies of the PBS-treated group (**F**), c-kit^−^ cell-treated group (**G**) and CT-treated group (**H**). Arrows indicate the vWF^+^ vessels; bar = 40 μm. *N* = 4–5 for each group. *P < 0.05, CT-treated group *versus*PBS-treated group.

The therapeutic effect of CT transplantation in promoting angiogenesis following MI was determined by comparing the presence of vWF^+^ vessels in the infarcted zone and the border zone. It was found that in the infarcted zone, the vessel density of CT-treated group was significantly larger than the PBS-treated group (*P* < 0.05). The vessel density of c-kit^−^ cell-treated group was larger than the PBS-treated group, but the difference was not statistically significant (*P* > 0.05; Fig. 4I_A-D_). In addition, in border zone, the vessel density of CT-treated group was significantly larger than the PBS-treated group (*P* < 0.05). The vessel density of c-kit^−^ cell-treated group was larger than the PBS-treated group, but the difference was not statistically significant (*P* > 0.05; Fig. 4II_E-H_).

### Implantation of CTs improved LV reconstruction following MI

**Fig. 5:**
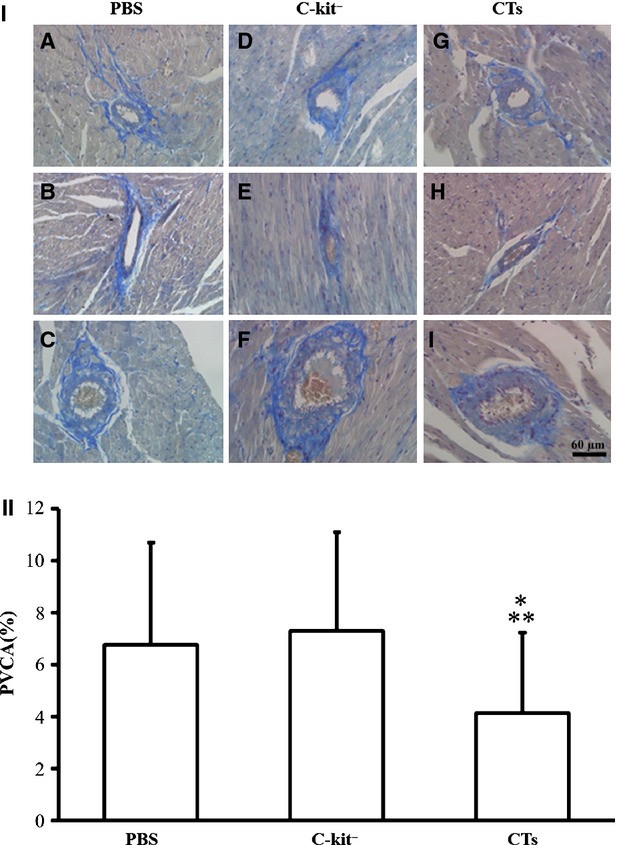
Transplantation of cardiac telocytes decreased collagen surrounding the peripheral vessels in non-infarcted zone. The perivascular collagen volume area (PVCA) of non-infarcted zone, 14 weeks after LAD ligation, was determined by analysis of collagen fibre staining. The PVCA of the CT-treated group was significantly reduced compared with the c-kit^−^ cell-treated group (*P* < 0.01) and PBS-treated group (*P* < 0.05). (I_A-C_) Representative morphology of PVCA in different size of vessel for the PBS-treated group; (I_D-F_) representative morphology of PVCA in different size of vessel for the c-kit^−^ cell-treated group; (I_G-I_) representative morphology of PVCA in different size of vessel for the CT-treated group. (II) Semi-quantification analysis of PVCA; bar = 60 μm. *N* = 4–5 for each group. **P < 0.01, CT-treated group *versus* c-kit^−^ cell-treated group; *P < 0.05, CT-treated group *versus*PBS-treated group.

**Fig. 6:**
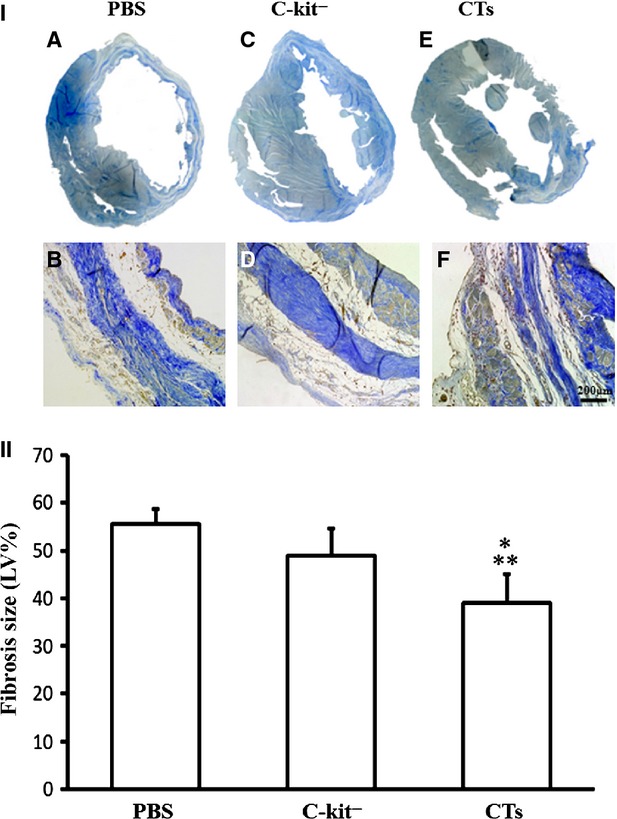
Transplantation of cardiac telocytes decreased collagen coverage in the infarcted zone. The collagen covered area of the infarcted zone (CAIZ), 14 weeks after LAD ligation, was determined and compared. The CAIZ of the CT-treated group was found to be significantly lower than the c-kit^−^ cell-treated group (*P* < 0.05) and PBS-treated group (*P* < 0.01). (I_A_) Representative cross dimension with collagen fibre staining for the PBS-treated group; (I_B_) representative morphology of CAIZ in infracted zone under higher power (20 × ) for the PBS-treated group; (I_C_) representative cross dimension with collagen fibre staining for the c-kit^−^ cell-treated group; (I_D_) representative morphology of CAIZ in infracted zone under higher power (20 × ) for the c-kit^−^ cell-treated group; (I_E_) representative cross dimension with collagen fibre staining for the CT-treated group; (I_F_) representative morphology of CAIZ in infracted zone under higher power (20 × ) for the CT-treated group. (II) Semi-quantification analysis of the CAIZ; bar = 200 μm. *N* = 4–5 for each group. **P < 0.01, CT-treated group *versus*PBS-treated group; *P < 0.05, CT-treated group *versus* c-kit^−^ cell-treated group.

**Fig. 7:**
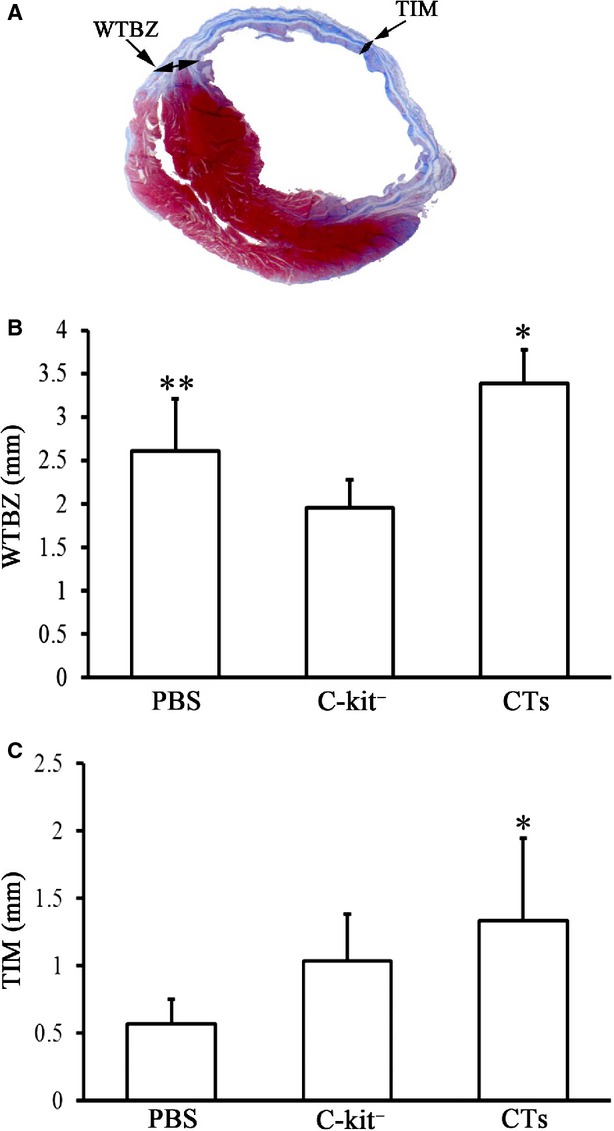
Transplantation of cardiac telocytes increased the thickness of infarcted myocardium and border zone wall. The thickness of the infarcted myocardium (TIM) and wall thickness of border zone (WTBZ) in the LV, 14-week post-LAD ligation, were determined by Masson*s trichrome staining. (**A**) Schematic drawing of the TIM and WTBZ. (**B**) The WTBZ of the CT-treated group was significantly higher than the c-kit^−^ cell-treated group and PBS-treated group (*P* < 0.01). The WTBZ of the c-kit^−^ cell-treated group was significantly lower than the PBS-treated group (*P* < 0.01). *N* = 4–5 for each group. *P < 0.01, CT-treated group *versus* c-kit^−^ cell-treated group and PBS-treated group. **P < 0.01 PBS-treated group *versus* c-kit^−^ cell-treated group. (**C**) The TIM of the CT-treated group was significantly wider than the PBS-treated group (*P* < 0.05). *N* = 4–5 for each group. *P < 0.05 CT-treated group *versus*PBS-treated group.

The possible therapeutic effect of CT transplantation in the reconstruction of the LV was evaluated. The parameters for assessing ventricular remodelling were as follows: (*i*) the peripheral vessels in the collagen area of non-infarcted zone (PVCA), (*ii*) the collagen area of infarcted zone (CAIZ), (*iii*) the wall thickness of border zone in LV (WTBZ) and (*iv*) the thickness of the infarcted myocardium in LV (TIM). These parameters obtained experimental and control hearts were compared. It was found that the PVCA in the CT-treated group was significantly lower than the c-kit^−^ cell-treated group and PBS-treated group (*P* < 0.01 CT-treated group *versus* c-kit^−^ cell-treated group; *P* < 0.05 CT-treated group *versus* PBS-treated group). However, there was no significant difference between the PVCA of c-kit^−^ cell-treated group and PBS-treated group (*P* > 0.05; Fig. 5). For infarcted zone, the CAIZ of the CT-treated group was significantly lower than that of the c-kit^−^ cell-treated group and PBS-treated group (*P* < 0.05 CT-treated group *versus* c-kit^−^ cell-treated group; *P* < 0.01 CT-treated group *versus* PBS-treated group). The CAIZ of the c-kit^−^ cell-treated group was lower than the PBS-treated group, but not statistically significant (*P* > 0.05; Fig. 6). The TIM of the CT-treated group was significantly wider than the PBS-treated group (*P* < 0.05), but not between the c-kit^−^ cell-treated group and PBS-treated group (*P* > 0.05). In addition, the WTBZ of the CT-treated group was significantly higher than the c-kit^−^ cell-treated group and PBS-treated group (*P* < 0.01). The WTBZ of the c-kit^−^ cell-treated group was significantly lower than the PBS-treated group (*P* < 0.01; Fig. 7).

## Discussion

In the present study, we examined the midterm effects of CT transplantation on experimentally induced MI rats. The results revealed that after 14 weeks post-CT implantation, the size of the infarct decreased significantly. In addition, the LV function of CT-treated infarct hearts was significantly improved, which was characterized by a higher EF of LV and lower diastolic and systolic diameters compared with the PBS-treated controls. Previously, we have reported that CT transplanted into the infarct site of MI rats for 2 weeks was able to decrease the infarct size and improve the LV function [32]. This finding combined with our present midterm results further support a protective role of CTs in the context of myocardial infarction. It also further suggests that the CT, its associated secretory factors and microvesicles might function as an important structural and functional unit of the myocardium to provide crucial beneficial effects needed for the repair of damaged myocardium.

The mechanism behind the beneficial effects of CT for treating MI is an intrigue issue. The results of our blood vessel density analysis demonstrated that CT transplantation could significantly increase the vessel density at both the heart infarct site and the border zone when compared with the control. This implies that one of beneficial effects of CT transplantation was enhanced angiogenesis. In fact, other studies have reported that CTs were able to secrete VEGF, express angiogenic-associated microRNAs and established direct nano-contacts with newly derived endothelial cells at the border zone of MI [29–31]. This implies that CTs are potentially very important for supporting angiogenesis in the repairing myocardium. This support might come in the form of CTs providing a structural and functional niche and microenvironment for the endothelial cells. Indeed, our double immuofluorescent staining revealed that there were significantly more c-kit^+^/CD34^+^ CTs distributed near and surrounding the vessels in infarct zone of CT-treated myocardium after 14 weeks transplantation compared with c-kit^−^ cell-treated group and PBS-treated group. Our results also demonstrated the beneficial effects imparted by the CT – such as promoting angiogenesis which in turn enhances the survival of transplanted CTs and the migration of CTs from the border zone into the infarct zone. It will benefit the reconstruction of CT network in the infarct zone, especially near the blood vessels. However, our experimental design could not prove that the c-kit^+^/CD34^+^ CTs found near the blood vessels of infarct zone were derived from the transplanted CTs or from CTs that have migrated from infarct border zone. Nevertheless, c-kit^+^/CD34^+^ CTs were also found around the vessels in the PBS-treated group and our prior study shows that CTs were not detectable in the infarct zone until 4 weeks after induced MI, but not in border zone [32]. This suggests that at least some of the c-kit^+^/CD34^+^ CTs found in the infarct zone of our CT-treated group were derived from the border zone. However, we cannot exclude the possibility which some of c-kit^+^/CD34^+^ CTs seen in our CT-treated myocardium were from transplanted CTs. Further study in future is needed to document this intriguing issue.

Myocardial fibrosis is one of key contributor to cardiac dysfunction after MI. It plays as a secondary response to the pathophysiologic remodelling of MI [36]. The pathological accumulation of extracellular matrices at the infarcted site might present a physical barrier that impairs the penetration of reparative stem/progenitor cells. Our fibrosis analysis showed that CT transplantation was also able to significantly decrease the fibrosis size in the infarcted zone and non-infarcted zone as compared with the control. This implies that another beneficial effect from the CT transplantation was to limit the extent of fibrosis and thereby improving the reconstruction of the LV. Indeed, comparison of the different reconstructed parameters between CT-treated and control groups revealed that the thickness of LV border zone wall and infarcted myocardium wall was greater in the CT-treated group. However, in present study, the molecular pathway underlined how the myocardial fibrosis was reduced after CT transplantation has not been answered. Further efforts are needed to establish CTs relationship with the pro-inflammatory cytokines, the activation of the myofibroblasts and macrophages, and the regulation of matrix metalloproteinases (enzymes involved in collagen degradation) at the infarct site. Indeed, ultrastructural analysis using electron microscope has revealed that the CT network could also integrate with the immune system (*via* the macrophages, mast cells), interstitium (*via* fibroblasts and extracellular matrix), vascular system (*via* endothelial cells and pericytes), nervous system (*via* Schwann cells), cardiac progenitor cells and cardiomyocytes in the myocardium [30]. For functional repair of the infarct myocardium, it is necessary to provide a microenvironment that could promote angiogenesis and support the invasion and development of cardiac progenitor cells. The unique characteristics of CTs, revealed in the present and previous studies [24–32], may play a positive contribution to this microenvironment and thereby enhancing the repair of the infarcted myocardium.

In summary, our results demonstrated that CT transplantation in MI was able to reduce the myocardial infarct size and improved cardiac function. The protective and beneficial effects from CTs were maintained for at least 14 weeks after transplantation. The cellular mechanism behind the beneficial effects of CT transplantation for MI was partially attributed to (*i*) an increase in cardiac angiogenesis, (*ii*) an improvement in the reconstruction of the CT network and (iii) a decrease in myocardial fibrosis. These combined effects were able to limit the infarct size which in turn improved the reconstruction of the LV and associated myocardial function in MI. The beneficial effects offered by CTs might form a potentially new type of cell-mediated therapy for treating MI. The therapy could be performed with CTs alone or in combination with stem cells (such as cardiac or bone marrow-derived stem cells).
